# Environmental and personal factors for osteoporosis or osteopenia from a large health check-up database: a retrospective cohort study in Taiwan

**DOI:** 10.1186/s12889-022-13938-8

**Published:** 2022-08-11

**Authors:** Ping-Chen Chung, Ta-Chien Chan

**Affiliations:** 1 Department of Dentistry, Puzi Hospital, Ministry of Health and Welfare, Chiayi, Taiwan; 2 Research Center for Humanities and Social Sciences, Academia Sinica, 128 Academia Road, Section 2, Taipei, Taiwan; 3 Institute of Public Health, School of Medicine, National Yang Ming Chiao Tung University, Taipei, Taiwan

**Keywords:** Osteoporosis, Health behaviors, Solar radiation, Walkability

## Abstract

**Background:**

Osteoporosis is an important public health issue in aging societies because of its associated morbidity, mortality, and decreased quality of life. The study aims to identify the association of low bone mineral density, including osteoporosis and osteopenia, with environmental and personal factors.

**Methods:**

The data of participants aged ≥ 20 years with multiple visits were obtained from a health check-up database in Taiwan from 2008 to 2016. Multivariable logistic regressions were performed to identify the selected factors associated with low bone mineral density for multiple visit data.

**Results:**

A total of 194,910 participants with 359,943 visits were included in this study. The prevalence of low bone mineral density (BMD) in the study population was 10.6% (*n* = 20.615). Older women, ever and current smokers (odds ratio (OR) = 1.04 [95% confidence interval (CI) = 1.01, 1.08]), or participants who were underweight (OR = 1.72 [1.64, 1.81]), consumed a vegetarian diet (OR = 1.32 [1.25, 1.39]), or had higher triglyceride levels (OR = 1.04 [1.01, 1.06]) were significantly associated with a higher risk of low BMD. Participants who had higher educational years (OR = 0.43 [0.41, 0.46]), higher physical activity (OR = 0.93 [0.89, 0.97]), appropriate sleep duration and better quality (OR = 0.98 [0.97, 0.99]), dairy intake (≥ 1 slice of yogurt or cheese/week, OR = 0.97 [0.95, 0.99]), higher uric acid (OR = 0.93 [0.91, 0.95]), higher walkability (OR = 0.997 [0.995,0.999]), and higher solar radiation exposure (OR = 0.997 [0.97,0.99]) were significantly associated with a lower risk of low BMD.

**Conclusion:**

Interventions in different directions, such as having better health behaviors, increasing sun exposure, and residing in a highly walkable environment, are beneficial for reducing the risk of low BMD.

## Background

Osteoporosis is characterized by low bone mass and microarchitectural deterioration of bone tissue. Osteoporosis is caused by a disorder in the equilibrium between bone resorption and formation [1]. A meta-analysis pooled different age groups and reported that the prevalence of osteoporosis in the world was 18.3% (95% CI 16.2–20.7), in women was 23.1% (95% CI 19.8–26.9), and in men was 11.7% (95% CI 9.6–14.1) [2]. The prevalence of osteoporosis among adults aged ≥ 50 years in Taiwan, estimated by the National Health Insurance Database in 2011, was 25.0%. In the same year, age ≥ 50 years accounted for 31.8% of the total population [3]. Low bone mineral density (BMD) is a risk factor for osteoporotic fractures, resulting in decreased quality of life, morbidity, disability, and mortality [1]. Osteoporosis-related fractures cause a high socioeconomic burden due to increased healthcare costs and care requirements [1]. In the United States, the annual average healthcare resource utilization and costs were $19,470 (standard deviation (SD): $36,894) for osteoporosis-related treatments and services [4]. In countries of the largest five countries of the European Union plus Sweden (EU6), the quality-adjusted life years (QALYs) related to fragility fractures in 2017 were 1.02 million (ages of 50 or more) and disability-adjusted life years (DALYs) related to fragility fractures in 2016 (ages of 50 to 100 years) were more than 2.6 million [5]. The total medical cost of major osteoporotic fractures per patient was USD 3,517 for men and USD 2,587 for women in the 2013 Taiwan National Health Insurance Research Database [6].

Osteoporosis is a common disease among the elderly. Therefore, prevention is a major public health issue worldwide. Previous studies have reported that the risk factors for osteoporosis include inadequate exercise, inadequate nutrition such as calcium and vitamin D, early menopause, smoking, and alcohol abuse [1]. Ultraviolet radiation (UVR) penetrates the skin and stimulates the conversion of 7-dehydrocholesterol to pre-vitamin D3 [7]. In addition, independent of vitamin D concentration, cumulative UVR exposure has a protective effect against osteoporotic fractures, particularly in women [8]. Several studies have shown that vitamin D and calcium are beneficial for skeletal health in adults [9]. However, the influence of dairy on different sites of BMD changes was heterogeneous and did not have a definitive conclusion. In a meta-analysis study, cheese and yogurt reduced the risk of osteoporotic fracture. However, no reduction was observed with milk [10].

Inadequate and long sleep durations are associated with osteoporosis [11]. However, the influence of sleep quantity on osteoporosis is inconsistent. Sasaki et al. pointed out that sleep disturbances related to sleep fragmentation are associated with osteoporosis, but sleep quantity is not obviously correlated with risk of osteoporosis [12]. Sleep fragmentation is involved in increased sympathetic nervous activity and adrenocortical activity [13]. Increased sympathetic nervous system activity results in increased bone resorption and decreased bone formation [14]. Physical activities, such as weight-bearing aerobic exercises and strength and resistance exercises, are reported to be useful for preventing bone loss among the elderly [15]. In addition to personal risk factors, environmental exposure, such as green space, has been positively associated with physical activity [16]. Accessibility to parks, open spaces, and green areas is related to healthy living and increasing accessibility can be used as a health-promoting strategy [17]. Neighborhood environments, such as parks or recreational areas promote residents’ physical activity and walking behaviors.

Previous studies have reported relationships between biochemical indicators, body composition, physical activity, nutritional status, and BMD [18 19]. However, almost all studies used cross-sectional data without long-term follow-up to explore factors and patterns of changes. This study aimed to identify the association of low BMD, including osteoporosis and osteopenia, with environmental exposure, such as walkability and solar radiation, dietary patterns, physical activity, and anthropometric and biochemical parameters, using a repeated health checkup database.

## Methods

### Data source and study participants

Participants’ data were obtained from a health check-up database maintained by the Mei Jau (MJ) Health Management Institution in Taiwan from 2008 to 2016. All participants completed a health examination, including a self-reported questionnaire about sociodemographic data, lifestyle, medication and dietary habits, anthropometric assessment, biochemical measurements, and bone mass measurement. Written informed consent from the participants was directly given to the Mei Jau (MJ) Health Management Institution in Taiwan. The researchers then applied the secondhand anonymized cohort database for further research purposes with approval from the ethical committee [20].

The ethical review was approved by the Institutional Review Board of Biomedical Science Research, Academia Sinica (AS-IRB-BM-21032). Individual-identifying data were removed, and participants remained anonymous throughout the study.

### Selection of the participants

**Fig. 1:**
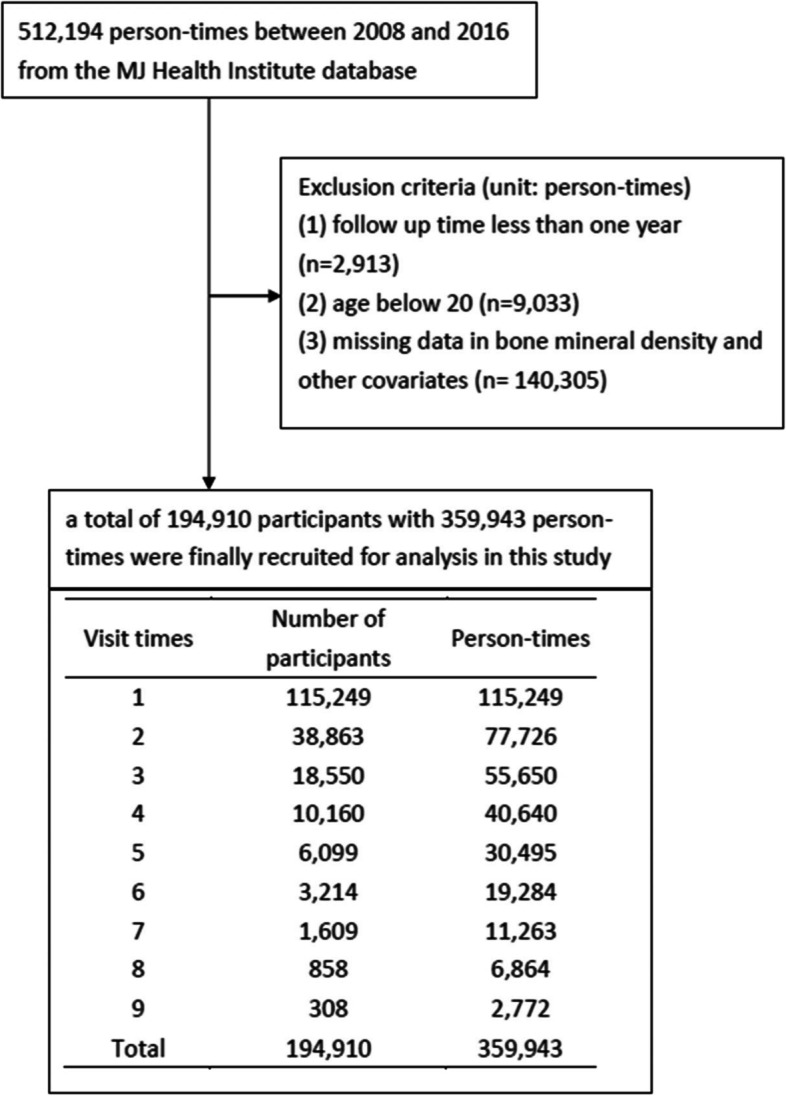
Flowchart of selecting participants

This study included data from 512,194 person-times between 2008 and 2016 from the MJ Health Management Institute database. After excluding a follow-up time of less than one year (*n* = 2,913), age < 20 years (*n* = 9,033), and missing data on BMD and other covariates (*n* = 140,305), 194,910 participants with 359,943 visits were finally included for analysis (Fig. 1).

We considered the following covariates as factors associated with low BMD: sociodemographic characteristics, lifestyle, sleep quality and duration, dietary, anthropometric data, biochemical data, walkability, and solar radiation.

### Sociodemographic characteristics, lifestyle, and sleep quality assessment

The sociodemographic characteristics included age, sex, and years of education (< 9, 9–14, 14–16, 16–18, and ≥ 18 years). Lifestyle characteristics included smoking status (0 = no, secondhand smoker; 1 = ever smoker and current smoker) and physical activity, assessed by intensity (light, moderate, heavy, or violent). Sleep scores, ranging from 2 (worst) to 9 (best), were calculated by adding sleep duration (range, 1–4) and quality category (range, 1–5). Participants reported sleep quality in the previous month and the average daily sleep duration. The categories of slept well, fell asleep but were easily awakened, excessive dreams while sleeping, difficulty falling asleep, and used sleeping pills or sedatives were assigned values of 5, 4, 3, 2, and 1, respectively. The categories of 4 h, 4–6 h, > 8 h, and 6–8 h from sleep duration were assigned values of 1, 2, 3, and 4, respectively [21].

### Dietary assessment and patterns

The numbers of servings and frequency of milk and dairy consumption were recorded using a self-reported questionnaire. Milk consumption was classified into two groups, including “none or < 1 cup a week” and “at least 1 cup a week” (1 cup is equivalent to 240 mL of fresh milk or yogurt, or 4 tablespoons of powdered milk). The frequency of dairy intake was divided into “none or less than a week” or”at least 1 slice of yogurt or cheese a week”. Moreover, the participants reported whether they were all-day vegetarians (0 = no; 1 = yes).

### Anthropometric and biochemical data

The body mass indexes (BMI) were classified as underweight, normal weight, overweight, or obesity (BMI < 18.5 kg/m^2^, 18.5 kg/m^2^ ≤ BMI < 24 kg/m^2^; 24 kg/m^2^ ≤ BMI < 27 kg/m^2^; and BMI ≥ 27 kg/m^2^, respectively) [22]. Central obesity was defined as a waist circumference > 80 cm for women and > 90 cm for men [23]. Participants who had long-term medication for controlling triglycerides (TG) or a TG value greater than or equal to 150 mg/dL were defined as having high TG levels [24]. Hyperuricemia was defined as a serum uric acid concentration ≥ 7.0 mg/dL in men and ≥ 6 mg/dL in women [25].

### Measurement of bone mineral density (BMD)

The lumbar spine’s BMD (g/cm^2^) was measured using a dual-energy X-ray absorptiometry densitometer (DXA, General Electric Lunar Health Care, DPX-L, USA). The BMD T-score is the number of SDs that compares the BMD value to a reference mean for young adults of the same sex. The low BMD group (osteopenia or osteoporosis) was defined by a T-score below − 1.0, and a T-score greater than or equal to − 1.0 was defined as the normal BMD group [1].

### Walkability

The positions with coordinates for each elementary school in 2015 were obtained from the Department of Statistics of the Ministry of Education. The park data were obtained from a point-of-interest database using the years 2008, 2010, 2012, and 2013. Walkability to parks and elementary schools was calculated as the sum of parks and elementary schools within a 1-km circular buffer (equivalent to approximately 10–15-min walk) of each participant’s geocoded coordinates of their residential addresses.

### Solar radiation

The Taiwan Climate Change Projection Information and Adaptation Knowledge Platform (TCCIP) offers daily solar radiation data with a resolution of 0.05° between 2015 and 2016. The TCCIP uses visible ray albedo and infrared brightness temperature data from geostationary satellites. The solar radiation received by the surface of the Taiwan region was estimated by the insolation inversion procedure, and nearly five-kilometer resolution grid data of the entire Taiwan region was established [26]. The digital terrain model (DTM) with a 20-m grid was derived from the Ministry of the Interior open data platform (https://data.gov.tw/dataset/35430). The solar radiation value was extracted by intersecting the participants’ residential coordinates and solar radiation data using QGIS. Because data before 2015 were not available, we replaced the values of these years with data from 2015.

### Statistical analysis

The sample was merged with walkability and solar radiation data by unique participants’ scrambled identification number and year of visit. The sample was divided into subgroups by sex and whether the women reached menopause. If there were no walkability or solar radiation data for the current year, used the data from the following year. Normality tests were performed using the Shapiro–Wilk test to determine the data distribution. The Mann–Whitney *U* test and chi-square test compared differences in BMD between the two groups, including low and normal BMD in baseline characteristics for continuous and categorical data, respectively. Univariate logistic regression was used to examine the association between baseline low BMD and baseline factors. Multivariable stepwise logistic regressions were used to select factors associated with low BMD at baseline for the total sample and each subgroup, including male, female, menopausal, and non-menopausal women. Multivariable logistic regressions with a generalized estimating equation (GEE) approach that used an unstructured correlation matrix were performed to identify the selected factors associated with low BMD at each visit. Statistical significance was set at *P* < 0.05. Statistical analysis was performed using R software, including MASS and geepack packages (R 3.0.2, The R Foundation for Statistical Computing, Vienna, Austria), and QGIS (version 3.4.7-Madeira).

## Results

**Fig. 2:**
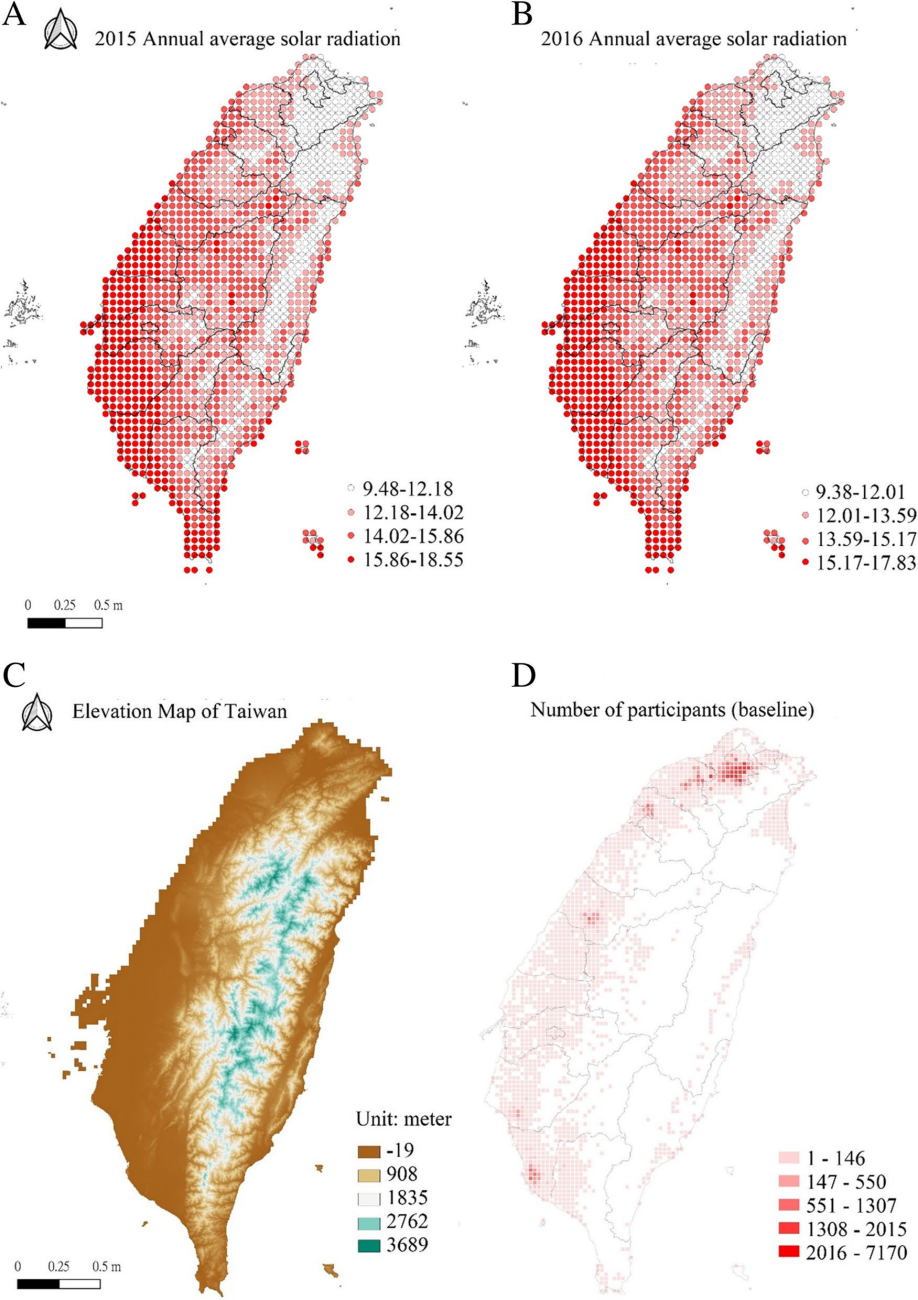
Spatial distribution of the selecting participants and their solar exposure. **A** 2015 Annual average solar radiation. **B** 2016 Annual average solar radiation. **C** Elevation map of Taiwan. **D** Number of participants (baseline)

Figure 2A and B show the annual average solar radiation distribution in 2015 and 2016, respectively. The average solar radiation was 14.02 with 1.84 in SD in 2015 and 13.59 with 1.58 in SD in 2016. High solar radiation occurs in the central and southern parts of the western coast. Relatively low solar radiation is located in the Central Mountain Range (Fig. 2C), the principal mountain range on Taiwan island running from island’s northeast to southwest. The number of participants at baseline was concentrated in the three major metropolitan areas of northern (*n* = 88,727; 45.5%), central (*n* = 8,603; 4.4%), and southern (12,284; 6.3%) Taiwan, respectively (Fig. 2D).

The baseline characteristics of the participants with low or normal BMD are presented in Table 1. The prevalence of low BMD in our study population was 10.6% (*n* = 20,615).Overall, the participants with low BMD were more likely to be female (58.6%), older (mea*n* = 53.7 years, SD = 14.5 years), and have low educational years (31.4%). The distribution of smoking status, physical activity, sleep duration and quality, dairy intake, vegetarian diet, BMI, waist circumference, triglycerides, uric acid, walkability, and solar radiation were significantly different between the low and normal BMD groups (*p* < 0.05). Among the male participants, the prevalence of heavy or violent exercise (11.2%), overweight (32.5%), obesity (20.4%), high triglycerides (30.8%), and hyperuricemia (37.1%) was high. Among female participants (shown in Table 2), 22.4% were menopausal. Menopausal women had no statistical difference regarding exposure between the normal and low BMD groups; women without menopause had no statistical difference in smoking status, physical activity, sleep duration and quality, milk intake, triglycerides, walkability, and exposure to solar radiation between the normal and low BMD groups.

**Table 1:** Descriptive statistic of the included variables by sex

	Total		Male			Female		
	Normal	low BMD	Normal	low BMD	Total	Normal	low BMD	Total
Characteristics	(*N* = 174,295)	(*N* = 20,615)	(*N* = 87,250)	(*N* = 8530)	(*N* = 95,780)	(*N* = 87,045)	(*N* = 12,085)	(*N* = 99,130)
**Age (years)**								
Mean (SD)	40.0 (11.4)	53.7 (14.5)	40.5 (11.8)	48.2 (14.4)	41.2 (12.3)	39.4 (11.0)	57.6 (13.2)	41.6 (12.8)
**Education**								
< 9 yr	9257 (5.3%)	6465 (31.4%)	3362 (3.9%)	1087 (12.7%)	4449 (4.6%)	5895 (6.8%)	5378 (44.5%)	11,273 (11.4%)
9 ~ 14 yr	36,814 (21.1%)	5550 (26.9%)	16,641 (19.1%)	2187 (25.6%)	18,828 (19.7%)	20,173 (23.2%)	3363 (27.8%)	23,536 (23.7%)
14 ~ 16 yr	35,084 (20.1%)	2892 (14.0%)	17,033 (19.5%)	1711 (20.1%)	18,744 (19.6%)	18,051 (20.7%)	1181 (9.8%)	19,232 (19.4%)
16 ~ 18 yr	64,336 (36.9%)	4016 (19.5%)	31,214 (35.8%)	2278 (26.7%)	33,492 (35.0%)	33,122 (38.1%)	1738 (14.4%)	34,860 (35.2%)
> = 18 yr	28,804 (16.5%)	1692 (8.2%)	19,000 (21.8%)	1267 (14.9%)	20,267 (21.2%)	9804 (11.3%)	425 (3.5%)	10,229 (10.3%)
**Smoking status**								
no, secondhand smoker	142,593 (81.8%)	17,471 (84.7%)	61,061 (70.0%)	5806 (68.1%)	66,867 (69.8%)	81,532 (93.7%)	11,665 (96.5%)	93,197 (94.0%)
ever smoker and current smoker	31,702 (18.2%)	3144 (15.3%)	26,189 (30.0%)	2724 (31.9%)	28,913 (30.2%)	5513 (6.3%)	420 (3.5%)	5933 (6.0%)
**Physical activity (intensity)**								
light exercise	125,164 (71.8%)	16,965 (82.3%)	51,407 (58.9%)	6143 (72.0%)	57,550 (60.1%)	73,757 (84.7%)	10,822 (89.5%)	84,579 (85.3%)
mean exercise	36,417 (20.9%)	2660 (12.9%)	25,823 (29.6%)	1651 (19.4%)	27,474 (28.7%)	10,594 (12.2%)	1009 (8.3%)	11,603 (11.7%)
heavy or violent exercise	12,714 (7.3%)	990 (4.8%)	10,020 (11.5%)	736 (8.6%)	10,756 (11.2%)	2694 (3.1%)	254 (2.1%)	2948 (3.0%)
**Sleep score**								
Mean (SD)	6.97 (1.33)	6.68 (1.54)	7.14 (1.29)	6.99 (1.36)	7.13 (1.30)	6.79 (1.34)	6.47 (1.62)	6.75 (1.38)
**Milk**								
no; < 1 cup/wk	95,666 (54.9%)	11,364 (55.1%)	49,457 (56.7%)	5068 (59.4%)	54,525 (56.9%)	46,209 (53.1%)	6296 (52.1%)	52,505 (53.0%)
> = 1 cup/wk	78,629 (45.1%)	9251 (44.9%)	37,793 (43.3%)	3462 (40.6%)	41,255 (43.1%)	40,836 (46.9%)	5789 (47.9%)	46,625 (47.0%)
**Dairy**								
no; < 1 slice of yogurt or cheese/wk	100,280 (57.5%)	14,107 (68.4%)	51,295 (58.8%)	5642 (66.1%)	56,937 (59.4%)	48,985 (56.3%)	8465 (70.0%)	57,450 (58.0%)
> = 1 slice of yogurt or cheese/wk	74,015 (42.5%)	6508 (31.6%)	35,955 (41.2%)	2888 (33.9%)	38,843 (40.6%)	38,060 (43.7%)	3620 (30.0%)	41,680 (42.0%)
**Vegetarian**								
Yes	4193 (2.4%)	1153 (5.6%)	1547 (1.8%)	279 (3.3%)	1826 (1.9%)	2646 (3.0%)	874 (7.2%)	3520 (3.6%)
No	170,102 (97.6%)	19,462 (94.4%)	85,703 (98.2%)	8251 (96.7%)	93,954 (98.1%)	84,399 (97.0%)	11,211 (92.8%)	95,610 (96.4%)
**BMI**								
normal	93,404 (53.6%)	12,064 (58.5%)	37,746 (43.3%)	4994 (58.5%)	42,740 (44.6%)	55,658 (63.9%)	7070 (58.5%)	62,728 (63.3%)
underweight	12,154 (7.0%)	1899 (9.2%)	1760 (2.0%)	624 (7.3%)	2384 (2.5%)	10,394 (11.9%)	1275 (10.6%)	11,669 (11.8%)
overweight	41,883 (24.0%)	4491 (21.8%)	29,052 (33.3%)	2032 (23.8%)	31,084 (32.5%)	12,831 (14.7%)	2459 (20.3%)	15,290 (15.4%)
obesity	26,854 (15.4%)	2161 (10.5%)	18,692 (21.4%)	880 (10.3%)	19,572 (20.4%)	8162 (9.4%)	1281 (10.6%)	9443 (9.5%)
**Waistline**								
central obesity	28,355 (16.3%)	3668 (17.8%)	17,903 (20.5%)	1121 (13.1%)	19,024 (19.9%)	10,452 (12.0%)	2547 (21.1%)	12,999 (13.1%)
normal	145,940 (83.7%)	16,947 (82.2%)	69,347 (79.5%)	7409 (86.9%)	76,756 (80.1%)	76,593 (88.0%)	9538 (78.9%)	86,131 (86.9%)
**Triglyceride**								
> = 150 mg/dl	36,338 (20.8%)	5022 (24.4%)	27,111 (31.1%)	2366 (27.7%)	29,477 (30.8%)	9227 (10.6%)	2656 (22.0%)	11,883 (12.0%)
normal	137,957 (79.2%)	15,593 (75.6%)	60,139 (68.9%)	6164 (72.3%)	66,303 (69.2%)	77,818 (89.4%)	9429 (78.0%)	87,247 (88.0%)
**Uric acid**								
hyperuricemia	43,642 (25.0%)	4579 (22.2%)	33,210 (38.1%)	2323 (27.2%)	35,533 (37.1%)	10,432 (12.0%)	2256 (18.7%)	12,688 (12.8%)
normal	130,653 (75.0%)	16,036 (77.8%)	54,040 (61.9%)	6207 (72.8%)	60,247 (62.9%)	76,613 (88.0%)	9829 (81.3%)	86,442 (87.2%)
**Walkability (park and elementary school; 1 km buffer)**								
Mean (SD)	5.88 (6.90)	5.25 (6.54)	5.75 (6.81)	5.26 (6.64)	5.71 (6.79)	6.00 (6.99)	5.25 (6.47)	5.91 (6.93)
**Solar radiation**								
Mean (SD)	13.5 (1.64)	13.6 (1.63)	13.5 (1.64)	13.6 (1.61)	13.5 (1.64)	13.5 (1.65)	13.6 (1.64)	13.5 (1.65)

**Table 2:** Descriptive statistic of the included variables by the menopausal status

	Menopause			Non-menopause		
	Normal	low BMD	Total	Normal	low BMD	Total
Characteristics	(*N* = 13,417)	(*N* = 8788)	(*N* = 22,205)	(*N* = 70,116)	(*N* = 2446)	(*N* = 72,562)
**Age (years)**						
Mean (SD)	56.9 (7.83)	62.5 (8.09)	59.1 (8.41)	35.9 (7.72)	38.9 (11.7)	36.0 (7.91)
**Education**						
< 9 yr	4591 (34.2%)	4715 (53.7%)	9306 (41.9%)	774 (1.1%)	190 (7.8%)	964 (1.3%)
9 ~ 14 yr	4978 (37.1%)	2572 (29.3%)	7550 (34.0%)	14,195 (20.2%)	532 (21.7%)	14,727 (20.3%)
14 ~ 16 yr	1643 (12.2%)	675 (7.7%)	2318 (10.4%)	15,771 (22.5%)	461 (18.8%)	16,232 (22.4%)
16 ~ 18 yr	1750 (13.0%)	679 (7.7%)	2429 (10.9%)	30,318 (43.2%)	997 (40.8%)	31,315 (43.2%)
> = 18 yr	455 (3.4%)	147 (1.7%)	602 (2.7%)	9058 (12.9%)	266 (10.9%)	9324 (12.8%)
**Smoking status**						
no, secondhand smoker	13,002 (96.9%)	8571 (97.5%)	21,573 (97.2%)	65,182 (93.0%)	2260 (92.4%)	67,442 (92.9%)
ever smoker and current smoker	415 (3.1%)	217 (2.5%)	632 (2.8%)	4934 (7.0%)	186 (7.6%)	5120 (7.1%)
**Physical activity (intensity)**						
light exercise	11,795 (87.9%)	7958 (90.6%)	19,753 (89.0%)	58,918 (84.0%)	2110 (86.3%)	61,028 (84.1%)
mean exercise	1294 (9.6%)	663 (7.5%)	1957 (8.8%)	8932 (12.7%)	270 (11.0%)	9202 (12.7%)
heavy or violent exercise	328 (2.4%)	167 (1.9%)	495 (2.2%)	2266 (3.2%)	66 (2.7%)	2332 (3.2%)
**Sleep score**						
Mean (SD)	6.49 (1.59)	6.39 (1.67)	6.45 (1.62)	6.85 (1.27)	6.78 (1.36)	6.85 (1.28)
**Milk**						
no; < 1 cup/wk	7222 (53.8%)	4534 (51.6%)	11,756 (52.9%)	37,214 (53.1%)	1329 (54.3%)	38,543 (53.1%)
> = 1 cup/wk	6195 (46.2%)	4254 (48.4%)	10,449 (47.1%)	32,902 (46.9%)	1117 (45.7%)	34,019 (46.9%)
**Dairy**						
no; < 1 slice of yogurt or cheese/wk	9250 (68.9%)	6477 (73.7%)	15,727 (70.8%)	37,661 (53.7%)	1385 (56.6%)	39,046 (53.8%)
> = 1 slice of yogurt or cheese/wk	4167 (31.1%)	2311 (26.3%)	6478 (29.2%)	32,455 (46.3%)	1061 (43.4%)	33,516 (46.2%)
**Vegetarian**						
Yes	723 (5.4%)	710 (8.1%)	1433 (6.5%)	1793 (2.6%)	102 (4.2%)	1895 (2.6%)
No	12,694 (94.6%)	8078 (91.9%)	20,772 (93.5%)	68,323 (97.4%)	2344 (95.8%)	70,667 (97.4%)
**BMI**						
normal	6707 (50.0%)	5107 (58.1%)	11,814 (53.2%)	46,826 (66.8%)	1458 (59.6%)	48,284 (66.5%)
underweight	324 (2.4%)	485 (5.5%)	809 (3.6%)	9724 (13.9%)	727 (29.7%)	10,451 (14.4%)
overweight	3720 (27.7%)	2093 (23.8%)	5813 (26.2%)	8465 (12.1%)	186 (7.6%)	8651 (11.9%)
obesity	2666 (19.9%)	1103 (12.6%)	3769 (17.0%)	5101 (7.3%)	75 (3.1%)	5176 (7.1%)
**Waistline**						
central obesity	3947 (29.4%)	2222 (25.3%)	6169 (27.8%)	6005 (8.6%)	139 (5.7%)	6144 (8.5%)
normal	9470 (70.6%)	6566 (74.7%)	16,036 (72.2%)	64,111 (91.4%)	2307 (94.3%)	66,418 (91.5%)
**Triglyceride**						
> = 150 mg/dl	3690 (27.5%)	2286 (26.0%)	5976 (26.9%)	5039 (7.2%)	187 (7.6%)	5226 (7.2%)
normal	9727 (72.5%)	6502 (74.0%)	16,229 (73.1%)	65,077 (92.8%)	2259 (92.4%)	67,336 (92.8%)
**Uric acid**						
hyperuricemia	3574 (26.6%)	1913 (21.8%)	5487 (24.7%)	6314 (9.0%)	168 (6.9%)	6482 (8.9%)
normal	9843 (73.4%)	6875 (78.2%)	16,718 (75.3%)	63,802 (91.0%)	2278 (93.1%)	66,080 (91.1%)
**Walkability (park and elementary school; 1 km buffer)**						
Mean (SD)	5.49 (6.66)	5.17 (6.39)	5.36 (6.56)	6.18 (7.09)	6.14 (6.98)	6.18 (7.09)
**Solar radiation**						
Mean (SD)	13.6 (1.70)	13.5 (1.62)	13.6 (1.67)	13.4 (1.62)	13.4 (1.56)	13.4 (1.61)

The unadjusted univariate associations between demographic data, health characteristics, lifestyle, and BMD groups among adult baseline visits are shown in Table 3. The results showed that female sex (odds ratio (OR) = 1.42, 95% CI = [1.38, 1.46]), older age (age OR = 1.082, 95% CI = [1.081, 1.084]), vegetarian diet (OR = 2.40, 95% CI = [2.25, 2.57]), central obesity (OR = 1.11, 95% CI = [1.07, 1.16]), higher triglycerides ≥ 150 mg/dl, OR = 1.22, 95% CI = [1.18, 1.26]) and higher solar radiation exposure (OR = 1.01, 95% CI = [1.01, 1.02]) were positively associated with low BMD, while longer educational years (≥ 18 years, OR = 0.08, 95% CI = [0.08, 0.09]), ever and current smoker (OR = 0.81, 95% CI = [0.78, 0.84]), heavy or violent exercise (OR = 0.58, 95% CI = [0.54, 0.62]), appropriate sleep duration and better sleep quality (OR = 0.86, 95% CI = [0.85, 0.87]), dairy intake (≥ 1 slice of yogurt or cheese/week, OR = 0.63, 95% CI = [0.61, 0.64]), obesity (BMI ≥ 27, OR = 0.62, 95% CI = [0.59, 0.65]), hyperuricemia (OR = 0.85, 95% CI = [0.83, 0.88]) and increased walkability (OR = 0.986, 95% CI = [0.984, 0.988]) were negatively associated with low BMD.

**Table 3:** Odds ratios for low BMD (lumbar spine T-score < -1 vs. T ≥ -1) for participants’ characteristics from univariate logistic regression models at the baseline

Characteristics	Odds Ratio	95%CI	*P*-value
**Age (years)**	1.082	(1.081, 1.084)	***
**Gender**			
Male	ref		
Female	1.42	(1.38, 1.46)	***
**Education**			
< 9 yr	ref		
9—14 yr	0.22	(0.21, 0.23)	***
14–16 yr	0.12	(0.11, 0.12)	***
16–18 yr	0.09	(0.09, 0.09)	***
> = 18 yr	0.08	(0.08, 0.09)	***
**Smoking status**			
no, secondhand smoker	ref		
ever smoker and current smoker	0.81	(0.78, 0.84)	***
**Physical activity (intensity)**			
light exercise	ref		
mean exercise	0.54	(0.52, 0.56)	***
heavy or violent exercise	0.57	(0.54, 0.61)	***
**Sleep score**	0.86	(0.85, 0.87)	***
**Milk**			
no; < 1 cup/wk	ref		
> = 1 cup/wk	0.99	(0.96, 1.02)	
**Dairy**			
no; < 1 slice of yogurt or cheese/wk	ref		
> = 1 slice of yogurt or cheese/wk	0.63	(0.61, 0.64)	***
**Vegetarian**			
No	ref		
Yes	2.40	(2.25, 2.57)	***
**BMI**			
normal	ref		
underweight	1.21	(1.15, 1.27)	***
overweight	0.83	(0.80, 0.86)	***
obesity	0.62	(0.59, 0.65)	***
**Waistline**			
normal	ref		
central obesity	1.11	(1.07, 1.16)	***
**Triglyceride**			
normal	ref		
> = 150 mg/dl	1.22	(1.18, 1.26)	***
**Uric acid**			
normal	ref		
hyperuricemia	0.85	(0.83, 0.89)	***
**Walkability (park and elementary school; 1 km buffer)**	0.986	(0.984, 0.988)	***
**Annual average solar radiation**	1.01	(1.01, 1.02)	***

After stepwise selection and fully adjusted GEE logistic regression analysis, the different selected variables in groups stratified by sex are presented in Table 4. Table 4 shows that older women, ever and current smokers (OR = 1.04, 95% CI = [1.01, 1.08]) or participants who were underweight (BMI < 18.5, OR = 1.72, 95% CI = [1.64, 1.81]), consumed a vegetarian diet (OR = 1.32, 95% CI = [1.25, 1.39]), or had higher triglyceride levels (≥ 150 mg/dl, OR = 1.04, 95% CI = [1.01, 1.06]) were significantly associated with a higher risk of low BMD. Participants with higher educational years (≥ 18 years, OR = 0.43, 95% CI = [0.41, 0.46]), higher physical activity (moderate exercise, OR = 0.93, 95% CI = [0.88, 0.97]), appropriate sleep duration and better quality (OR = 0.98, 95% CI = (0.97, 0.99)), dairy intake (≥ 1 slice of yogurt or cheese/week, OR = 0.97, 95% CI = [0.95,0.99]), hyperuricemia (OR = 0.93, 95% CI = [0.91, 0.95]), higher walkability (OR = 0.997, 95% CI = [0.995, 0.999]), and higher solar radiation (OR = 0.98, 95% CI = [0.97, 0.99]) were significantly associated with a lower risk of low BMD. Among male participants, those who consumed milk (≥ 1 cup/week, OR = 0.96, 95% CI = [0.93, 0.99]) were less likely to have low BMD; however, dairy consumption and triglycerides level were not significantly associated with BMD. Among male participants, ever and current smokers were significantly associated with low BMD (OR = 1.05, 95% CI = [1.01, 1.09]). Among the female participants, menopausal women (OR = 3.81, 95% CI = [3.56,4.07]) had a higher risk of having low BMD, but smoking status, sleep duration and quality, dairy consumption, triglycerides, and walkability were not significantly associated with BMD. Among menopausal woman (shown in Table 5), those who were on a vegetarian diet (OR = 1.25, 95% CI = [1.14, 1.38]) and had longer sleep duration and better quality (OR = 1.01, 95% CI = [1.00,1.03]) were more likely to have low BMD. However, central obesity (OR = 0.90, 95% CI = [0.85, 0.95]), hyperuricemia (OR = 0.87, 95% CI = [0.83,0.90]), higher physical activity (moderate exercise, OR = 0.94, 95% CI = [0.88, 0.99]), and longer solar radiation exposure (solar radiation OR = 0.97, 95% CI = [0.96, 0.99]) were less likely to have low BMD risk in menopausal women.

**Table 4:** Adjusted multivariable logistic regression with GEE approach coefficients for participants' characteristics and low BMD risk factors among adults aged ≥ 20 years stratified by sex in Taiwan

		Total		Sex					
				Male			Female		
Characteristics	OR	95% CI	*P-*value	OR	95% CI	*P-*value	OR	95% CI	*P-*value
**Age (years)**	1.07	(1.07, 1.07)	***	1.04	(1.04, 1.04)	***	1.08	(1.08, 1.08)	***
**Education**									
< 9 yr	ref			ref			ref		
9 ~ 14 yr	0.56	(0.54, 0.58)	***	0.83	(0.76, 0.90)	***	0.62	(0.59, 0.66)	***
14 ~ 16 yr	0.45	(0.42, 0.47)	***	0.76	(0.69, 0.82)	***	0.51	(0.48, 0.55)	***
16 ~ 18 yr	0.44	(0.42, 0.46)	***	0.69	(0.63, 0.75)	***	0.58	(0.54, 0.62)	***
> = 18 yr	0.43	(0.41, 0.46)	***	0.66	(0.6, 0.72)	***	0.51	(0.46, 0.57)	***
**Smoking status**									
no, secondhand smoke	ref			ref			NA		
ever smoker and current smoker	1.04	(1.01, 1.08)	*	1.05	(1.01, 1.09)	*	NA		
**Physical activity (intensity)**									
light exercise	ref			ref			ref		
mean exercise	0.91	(0.89, 0.94)	***	0.87	(0.84, 0.90)	***	0.93	(0.88, 0.97)	**
heavy or violent exercise	0.93	(0.89, 0.97)	**	0.85	(0.81, 0.90)	***	0.91	(0.83, 1.00)	
**Sleep score**	0.98	(0.97, 0.99)	***	0.98	(0.97, 0.99)	***	NA		
**Milk**									
no; < 1 cup/wk	NA			ref			NA		
> = 1 cup/wk	NA			0.96	(0.93, 0.99)	**	NA		
**Dairy**									
no; < 1 slice of yogurt or cheese/wk	ref			NA			ref		
> = 1 slice of yogurt or cheese/wk	0.97	(0.95, 0.99)	***	NA			0.97	(0.94, 1)	
**Vegetarian**									
No	ref			ref			ref		
Yes	1.32	(1.25, 1.39)	***	1.26	(1.15, 1.39)	***	1.29	(1.2, 1.4)	***
**BMI**									
normal	ref			ref			ref		
underweight	1.72	(1.64, 1.81)	***	2.21	(2, 2.45)	***	2.19	(2.04, 2.34)	***
overweight	0.65	(0.63, 0.67)	***	0.63	(0.6, 0.65)	***	0.61	(0.58, 0.64)	***
obesity	0.48	(0.46, 0.5)	***	0.45	(0.43, 0.48)	***	0.41	(0.39, 0.44)	***
**Waistline**									
normal	ref			NA			NA		
central obesity	0.99	(0.96, 1.03)		NA			NA		
**Triglyceride**									
normal	ref			NA			NA		
> = 150 mg/dl	1.04	(1.01, 1.06)	**	NA			NA		
**Uric acid**									
normal	ref			ref			ref		
hyperuricemia	0.93	(0.91, 0.95)	***	0.89	(0.87, 0.92)	***	0.86	(0.82, 0.89)	***
**Walkability (park and elementary school; 1 km buffer)**	1.00	(0.995, 0.999)	***	0.99	(0.99, 0.996)	***	1.00	(0.996, 1.002)	
**Solar radiation**	0.98	(0.97, 0.99)	***	0.98	(0.97, 0.99)	**	0.97	(0.96, 0.99)	***
**Menopause**									
No	NA			NA			ref		
Yes	NA			NA			3.81	(3.56, 4.07)	***

**Table 5:** Adjusted multivariable logistic regression with GEE approach coefficients for participants' characteristics and low BMD risk factors among adults aged ≥ 20 years stratified by the menopausal status in Taiwan (continue)

	Menopausal	women		Non-menopausal	women	
Characteristics	OR	95%CI	*P-*value	OR	95%CI	*P-*value
**Age (years)**	1.10	(1.09, 1.10)	***	1.05	(1.04, 1.06)	***
**Education**						
< 9 yr	ref			ref		
9 ~ 14 yr	0.73	(0.69, 0.78)	***	0.23	(0.19, 0.27)	***
14 ~ 16 yr	0.63	(0.58, 0.69)	***	0.19	(0.16, 0.22)	***
16 ~ 18 yr	0.62	(0.57, 0.67)	***	0.22	(0.19, 0.27)	***
> = 18 yr	0.56	(0.48, 0.66)	***	0.19	(0.16, 0.24)	***
**Smoking status**						
no, secondhand smoker	NA			NA		
ever smoker and current smoker	NA			NA		
**Physical activity (intensity)**						
light exercise	ref			NA		
mean exercise	0.93	(0.88, 0.99)	*	NA		
heavy or violent exercise	0.94	(0.83, 1.06)		NA		
**Sleep score**	1.01	(1.00, 1.03)	*	0.98	(0.96, 1.00)	
**Milk**						
no; < 1 cup/wk	NA			NA		
> = 1 cup/wk	NA			NA		
**Dairy**						
no; < 1 slice of yogurt or cheese/wk	ref			NA		
> = 1 slice of yogurt or cheese/wk	0.97	(0.93, 1.00)		NA		
**Vegetarian**						
No	ref			ref		
Yes	1.25	(1.14, 1.38)	***	1.26	(1.06, 1.51)	**
**BMI**						
normal	ref			ref		
underweight	1.64	(1.48, 1.82)	***	2.22	(2.03, 2.43)	***
overweight	0.67	(0.64, 0.70)	***	0.56	(0.51, 0.63)	***
obesity	0.48	(0.44, 0.52)	***	0.33	(0.27, 0.41)	***
**Waistline**						
normal	ref			ref		
central obesity	0.90	(0.85, 0.95)	***	1.10	(0.95, 1.29)	
**Triglyceride**						
normal	ref			ref		
> = 150 mg/dl	0.97	(0.93, 1.01)		1.09	(0.98, 1.20)	
**Uric acid**						
normal	ref			ref		
hyperuricemia	0.87	(0.83, 0.90)	***	0.99	(0.89, 1.09)	
**Walkability (park and elementary school; 1 km buffer)**	0.997	(0.994, 1.001)		NA		
**Solar radiation**	0.97	(0.96, 0.99)	***	0.98	(0.96, 1.00)	
**Menopause**						
No	NA			NA		
Yes	NA			NA		

*NA*: The variable was not selected in the baseline by the stepwise logistic regression

*abbreviation*: *OR* Odds ratio

^*^: *P* value < 0.05, **: *P* value < 0.01; ***: *P* value < 0.001

## Discussion

This health check-up cohort found a significantly strong effect on the low BMD of some personal features, such as female, older age, low education level, ever and current smokers, low physical activity intensity, and low BMI. In addition, compared to that of previous cross-sectional studies, this longitudinal study confirmed that shorter sleep duration and poor sleep quality, low dairy consumption, vegetarian diet, high triglyceride levels, low uric acid levels, low walkability, and low solar radiation exposure were associated with low BMD.

A higher education level was positively correlated with preventing osteoporosis by having more opportunities to have adequate knowledge and a good attitude toward preventing osteoporosis [27].

In univariate logistic regression, ever and current smokers were shown to be less likely to have low BMD at baseline, possibly owing to the effect of sex and other unadjusted confounders. In the total sample and male subgroup, ever and current smokers were more likely to have low BMD in multivariable logistic regressions using the GEE approach. In the female subgroup, smoking status was not selected as an influencing variable to put into multivariable logistic regression. The effect of smoking on BMD differed between men and women. Among men, current and recent smokers showed a lower BMD than that of non-smokers in a cohort of older men and women with over 40 years of follow-up. However, among women who did not use estrogen, no significant association was found between smoking and BMD [28].

Regular physical activity promotes bone health during aging. Compared with light-intensity exercises such as strolling, Tai Chi, playing golf, and gardening, moderate-intensity exercises such as badminton, brisk walking, and dancing were significantly negatively correlated with osteoporosis. However, heavy or violent intensity exercises such as swimming and running were not significant in the present study. For males, among light-, moderate-, and heavy- or violent-intensity exercises, the latter is the most effective in preventing osteoporosis. Controlled trials suggest that moderate-to high-intensity exercise, especially combined resistance and impact training, provide a positive stimulus to lumbar spine BMD and that low-intensity exercise is relatively ineffective [29]. However, not only does intensity affect BMD, but exercise volume, duration, and frequency are also related to BMD [15]. According to the different types of exercise, the amount of BMD increase varies in different parts, such as the lumbar spine, femoral neck, and total hip [15].

It has been reported that too short or too long sleep duration, taking a daytime nap, and poor sleep quality increase the risk of osteoporosis [11 30]. A large-scale cross-sectional study revealed that women aged over 50 years with sleep duration < 5 h/day had a higher risk of osteoporosis (OR = 7.35; 95% CI 3.43–15.71), and participants with poor sleep quality had 5.57 (95% CI 1.60–19.41) odds of osteoporosis [31].

Vegetarians, especially vegans, have a lower BMD and a higher fracture risk than non-vegetarians [32]. In Taiwan, soy products, such as soy milk and tofu, are typical diets rich in proteins and isoflavones. Soy protein and isoflavones may play a role in the retardation of bone loss [33]. Increasing the knowledge of nutrients and elevating the quality of vegetarian and vegan diets can reduce the decline in BMD.

Dairy products are rich in protein, calcium, phosphorus, magnesium, and zinc, and are beneficial for bone production. Regardless of the type of dairy product, there was a positive association with the reduced risk of low BMD.

In our study, increasing BMI was inversely related to low BMD. This conclusion is similar to that of previous studies [34]. Two mechanisms have been proposed for this phenomenon. First, a higher BMI enhances bone strength to load heavier mechanical loading of total body weight in early adulthood [35]. Second, the production of adipose tissue, such as adipokines, estrogen, leptin, and interleukin-6, can regulate metabolic processes in bone tissue through different mechanisms [35].

An inverse relationship between the waist circumference and lumbar BMD among middle-aged men and premenopausal women is revealed in a four-wave of the National Health and Nutrition Examination Survey from 2011 to 2018 in United States [36]. Increasing fat mass has a negative correlation with bone mass when excluding the mechanical loading effect of body weight, and waistlines can play a role in the evaluation of abdominal fat accumulation [35].

Consistent with previous studies, an inverse relationship between triglycerides and BMD was observed [37]. A Mendelian randomization study reported a causal weak negative effect of triglycerides on BMD (effect size (standard error) = 0.013(0.005)) [37].

Uric acid plays a paradoxical role in osteoporosis. Uric acid acts as a pro-oxidant in the cell and an antioxidant, mainly in plasma [38]. Pro-oxidants are related to reduced BMD, and antioxidants exert protective effects against osteoporosis. A recent review concluded that normal or high uric acid levels are associated with decreased bone resorption and increased bone formation; however, hyperuricemia has the opposite effect [39].

In addition, we found that walkability was negatively correlated with low BMD. Promoting the convenience of walkability is a factor that prevents the acceleration of bone resorption. The attractiveness of open spaces and neighborhood-built environments, such as parks or playground equipment, is essential to individuals walk and elevate their physical activity behaviors. Sugiyama et al. pointed out that nearest neighborhood open spaces within a 1.6-km radius from residences are likely to encourage recreational walking [16]. The outdoor space of elementary schools is open for public use outside of school time in Taiwan. Many Taiwanese people, especially children and elders, like to exercise in parks or schools. The present study found that the number of schools and parks within a 1 km buffer of residence was associated with a reduced risk of osteoporosis.

Vitamin D is mainly acquired from sun exposure, and small quantities of vitamin D are absorbed from the diet [7]. Vitamin D is transferred to 1,25(OH)_2_D_3_ in the circulation, and 1,25(OH)_2_D_3_ can induce bone mineralization. Taiwan is located across the tropics and subtropics, between 20° N and 25° N. The effect of solar radiation on BMD was observed in the present study. Sunlight exposure depends on geographic location, time of day, season, climatic conditions, and air pollution. Leal et al. showed that vitamin D_3_ production in São Paulo (LAT 23° S) was 20.1% (95% CI 12.9–27.2) lower than that in Fortaleza (LAT 3° S) over a year in vitro experiment [7]. Farrar et al. indicated sun-exposure guidelines on vitamin D acquisition, which is noontime unshaded summer sunlight exposure of 30 min at 30°N three times per week with sufficient skin surface area exposed [40].

Menopause has an important impact on the endocrine, skeletal, cardiovascular, immune, and genitourinary systems [41]. After menopause, the bone-sparing effect of estrogen reduces bone resorption by inhibiting osteoclast activity. When estrogen deficiency increases, the release of bone-resorbing cytokines stimulates osteoclast activity [42]. Postmenopausal plasma estrogen is mainly produced and metabolized by adipose tissue. Increased plasma concentrations of estrogens are observed with greater body weight [41] and are related to abdominal fat deposition. The concentration of estrogen is also affected by solar radiation. Relevant to the present study, changes in uric acid, triglycerides, and sleep are related to changes in sex hormones, especially in the postmenopausal period. One pathway associated with uric acid regulation is conjugated estrogen, which increases renal clearance of plasma uric acid [43]. In general, plasma uric acid concentration is higher in men than in women, and postmenopausal women are lower than premenopausal women [43]. Loss of estradiol is associated with increased triglyceride levels, which was also observed in previous studies. However, other factors influence triglyceride levels, such as body weight [44]. Aging, endocrine, and psychological status affect sleep disturbances, including trouble falling asleep and frequent nocturnal awakenings during the menopausal transition [45]. However, some studies have suggested no direct correlation between sleep problems and hormonal changes during menopause [46]. In our study, appropriate sleep duration and good sleep quality were positively associated with low BMD, but only in postmenopausal women.

The strength of this study is its large sample size and repeated health check-up data with a comprehensive assessment. The longest follow-up period was nine years. One limitation of our study was that self-reported questionnaires may have led to recall bias, especially the amount and frequency of food consumption. Second, despite the large sample size, the prevalence of low BMD (10.66%) in the study population was relatively low. Because the study used Mei Jau health exam data, in which participants were healthier, people with osteoporosis or osteopenia may be less likely to attend health examination and more likely to seek medical resources. Third, the solar exposure of each participant was not quantified because of data limitations. Fourth, there may be some unmeasured factors that are potential confounders, such as estrogen level, mood symptoms, and objective sleep data. Fifth, the generalization of our findings to whole Taiwanese population is also limited because the studied cohort might have had a higher socioeconomic status than the general population [47]. In the future, we will explore the relationship between sleep status and BMD during the peri- and postmenopausal periods after adjusting for psychological factors and hormone levels.

## Conclusions

Interventions in different directions, including regulating physical activity and dairy intake, maintaining a normal BMI, and appropriate sleep habits, can prevent low BMD. Establishing a neighborhood physical environment and adding pedestrian friendliness would result in higher physical activity levels in a highly walkable environment. Based on population density and the availability of destinations, creating walkable environments through policymaking could be considered a measure for promoting citizens’ daily physical activities.

## Data Availability

The data that support the findings of this study are available from MJ Health Research Foundation but restrictions apply to the availability of these data, which were used under license for the current study, and so are not publicly available. Data are however available from the authors upon reasonable request and with permission of MJ Health Research Foundation.
